# Surround Modulation Properties of Tectal Neurons in Pigeons Characterized by Moving and Flashed Stimuli

**DOI:** 10.3390/ani12040475

**Published:** 2022-02-15

**Authors:** Xiaoke Niu, Shuman Huang, Minjie Zhu, Zhizhong Wang, Li Shi

**Affiliations:** 1 Henan Key Laboratory of Brain Science and Brain-Computer Interface Technology, School of Electrical Engineering, Zhengzhou University, Zhengzhou 450001, China; niuxiaoke@zzu.edu.cn (X.N.); schuman@stu.zzu.edu.cn (S.H.); zhumj@zzu.edu.cn (M.Z.); wzz1982@zzu.edu.cn (Z.W.); 2 Department of Automation, Tsinghua University, Beijing 100084, China

**Keywords:** surround suppression, extra-classical receptive field, optic tectum, size tuning

## Abstract

**Simple Summary:**

Surround modulation is a basic visual attribute of sensory neurons in many species and has been extensively characterized in mammal primary visual cortex, lateral geniculate nucleus, and superior colliculus. Little attention has been paid to birds, which have a highly developed visual system. We undertook a systematic analysis on surround modulation properties of tectal neurons in pigeons (*Columba livia*). This study complements existing studies on surrounding modulation properties in non-mammalian species and deepens the understanding of mechanisms of figure–background segmentation performed by avians.

**Abstract:**

Surround modulation has been abundantly studied in several mammalian brain areas, including the primary visual cortex, lateral geniculate nucleus, and superior colliculus (SC), but systematic analysis is lacking in the avian optic tectum (OT, homologous to mammal SC). Here, multi-units were recorded from pigeon (*Columba livia*) OT, and responses to different sizes of moving, flashed squares, and bars were compared. The statistical results showed that most tectal neurons presented suppressed responses to larger stimuli in both moving and flashed paradigms, and suppression induced by flashed squares was comparable with moving ones when the stimuli center crossed the near classical receptive field (CRF) center, which corresponded to the full surrounding condition. Correspondingly, the suppression grew weaker when the stimuli center moved across the CRF border, equivalent to partially surrounding conditions. Similarly, suppression induced by full surrounding flashed squares was more intense than by partially surrounding flashed bars. These results suggest that inhibitions performed on tectal neurons appear to be full surrounding rather than locally lateral. This study enriches the understanding of surround modulation properties of avian tectum neurons and provides possible hypotheses about the arrangement of inhibitions from other nuclei, both of which are important for clarifying the mechanism of target detection against clutter background performed by avians.

## 1. Introduction

The visual response to classical receptive field (CRF) stimuli can be modulated by the extra-classical receptive field (eCRF), where stimulating alone cannot elicit spikes [1,2,3]. This phenomenon is called “surround modulation”, which is generally suppressive [4,5,6,7,8,9,10,11,12,13] rather than facilitative [14,15,16,17,18], and is related to visual saliency representation and figure–ground segregation [9,19]. The fundamental nature of surround modulation has been fairly described in mammal primary visual cortex (V1), lateral geniculate nucleus (LGN), and superior colliculus (SC), and the existing studies have concluded that the surround modulation in V1 and LGN was selective to visual features, such as orientation and spatial frequency [20,21]. In addition, the response modulations in SC were reported to be more noticeable to direction contrast compared to phase, temporal frequency, and static orientation contrast [22]. Subsequent studies further showed that the surround suppression could sharpen the orientation tuning [23] and enhance the single neuron’s orientation selectivity [24,25], as well as the connection strength and the network structural properties among a local neuronal population in V1 [26]. The most recent work further revealed that dynamic surround suppression presented in SC was affected by temporal context (adaptation) [27]. By contrast, relatively fewer studies have been devoted to non-mammalian species, especially birds, who have evolved highly advanced visual systems [28,29,30,31]. Intriguingly, the geniculo-striate (homologous to thalamofugal in birds) pathway plays a dominant role in supporting visual acuity in mammals, whereas the tectofugal pathway is dominant in birds [30]. In addition, pigeons have been widely used as an animal model in the majority of visual neuroscience studies [29]. As far as we know, there lacks detailed and systematic analysis of surround modulation properties of tectal neurons in pigeons.

The surround modulation properties reported in mammals are almost related to the nature of neurons, which are selective to orientation or spatial frequency [22,32,33]. In avians, neurons in OT mostly respond to motion [34,35] or change in luminance [36,37]. Only a small number of neurons present as direction selective and none preferred orientation [38,39,40]. An earlier study on surround modulation has reported that the motion evoked tectal response was modulated by large moving background [41]. The suppression was strongest when the moving direction of the center was the same as that of the background [42,43]. Tectal neurons also responded to spatial contrasting stimuli, when the contrast between center and background was the direction of motion rather than the orientation, and when the center was looming and the background receding but not when the center was receding and the background looming [44]. The surround suppression upon tectal response was less when the surrounding elements all moved in one direction (homogeneously moving), compared with nonhomogeneously moving elements [45]. A recent work further reported that the tectal neuronal response to a single small static flashed bar could also be inhibited by a group of surrounding flashed bars, and the suppression was strong when the luminance between the center and surroundings was the same, but less than motion direction contrasting paradigm when the motion direction between the center and surrounding was the same [46]. Since tectal neurons respond to both motion and flashed stimuli, both of which can also induce surrounding modulation, it is unclear whether there were similarities and differences between surrounding modulation properties derived from motion and flashed paradigms. In addition, since most tectal neurons have no motion direction selectivity and none preferred any orientation, it could be implied that bars may evoke comparable tectal neuronal responses with squares (with no orientation) in either flashed or moving paradigms. Nevertheless, there lacks experimental examination, and it is still unknown whether there were any differences between the surrounding modulation induced by bars and squares either in motion or flashed paradigm. Furthermore, when the flashed bar is located at the CRF center and its short side equals the diameter of CRF, the stimulation with bars is equivalent to partial surrounding mode. For the squares, when its side length equals the long side of the bar, the stimulation with squares just corresponds to the full surrounding mode. In the moving paradigm, the full and partial surrounding modes, respectively, correspond to when the square crosses the CRF center and border. Taken together, it would help to make clear whether the surrounding modulation that exists in OT (dominated by inhibition) was full surrounding or local lateral by comparing the difference and similarity between surround modulation by bars and squares either in motion or flashed paradigm. The above answers would help to further understand the surround suppression, and this knowledge will provide critical insights into understanding the mechanism of object detection in complex scenes.

To answer the above questions, we recorded multi-units from the middle layer in anesthetized pigeons OT with multi-electrode array (MEA), and designed visual stimuli, including different sizes of flashed squares and bars, as well as moving stimuli of the same shapes and sizes. We analyzed and compared the neuronal data during responses to different types of the visual stimuli, hoping to systematically explore the surround modulation properties of tectal neurons in pigeons. Together, the insights gained in this study will help to further understand the surround suppression and help to further understand the mechanism of target detection against clutter background performed by avians.

## 2. Materials and Methods

### 2.1. Animal Preparation

Our data were recorded from 17 pigeons (Columba livia, either sex, weighing 300–500 g) maintained by the Animal Center of Zhengzhou University. The study was conducted according to the guidelines of the National Institutes of Health. All experiments were approved by the Animal Care and Use Committee of Zhengzhou University (No SYXK 2019-0002).

The experimental preparation was consistent with our previous study [46]. Each pigeon was anesthetized with urethane (1.8–2.2 g/kg body weight) and restrained in a stereotaxic apparatus (model ST-5ND-B; Chengdu Instrument, Chengdu, China). A small dorso-lateral tectum region on the left side was exposed for craniotomy [47]. The right eye was held open by removing the nictitating membrane and eyelids with surgical scissors, and the left eye was covered. Multi-units activities were recorded with an MEA (the same type with our previous study [46]) that consisted of 16 polyimide-insulated platinum/iridium microwires (Clunbury Scientific, Bloomfield Hills, MI, USA), which were arranged in four rows with four wires in each row (electrode spacing = 550 µm; row spacing = 250 µm; impedance = 20–50 kΩ). The recording sites were verified by a following histological reconstruction.

The experimenter assessed the pigeon’s eye movements by intermittently monitoring the anesthetized pigeons during data recording, and no eye movement was observed [48]. Furthermore, the receptive field (RF) at each recording site was remapped at intervals of 30 min by the experimenter, and those without RF location shifts were used to carry out further visual stimulation experiments.

### 2.2. Visual Stimuli and Electrophysiological Recordings

**Figure 1:**
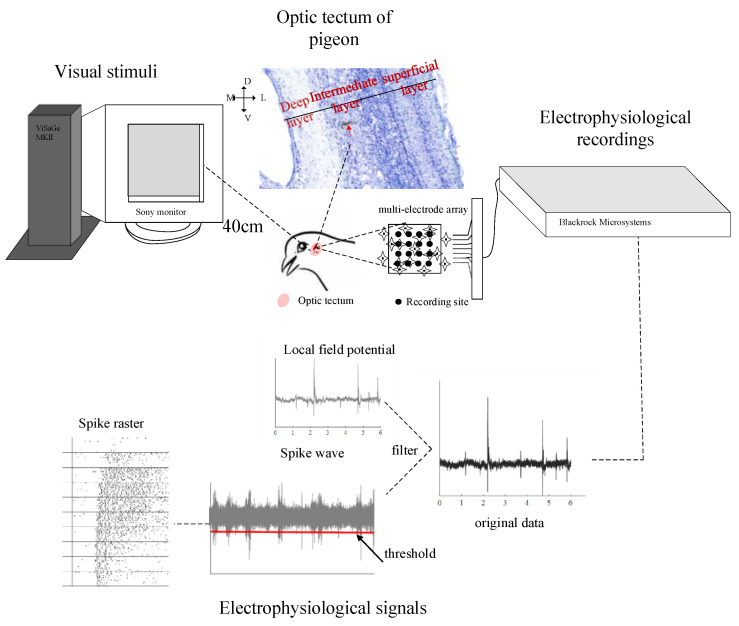
Schematic illustration of the experiment. Pigeons were anesthetized and restrained in a stereotaxic apparatus. Visual stimuli were generated with the ViSaGe MKII visual stimulus generator and displayed on a cathode ray tube monitor positioned 40 cm in front of each pigeon’s right eye. The neuronal signals were synchronously collected with a cerebus system. The spikes were detected by thresholding the band-pass filtered (Second-order Butterworth) raw signals between 250 and 5 kHz with a sampling rate of 30 kHz. The lower left presents an example of spike trains of 20 repeats under different stimuli. The vertical line indicates the onset of each stimulus and the horizontal line separates different stimuli. Each black dot represents a spike.

Visual stimuli were generated with the ViSaGe MKII visual stimulus generator (Cambridge Research Systems, Rochester, England) and presented on a gamma-calibrated Sony monitor (monitor size: 300 × 400 mm; resolution: 480 × 640 pixels; frame rate: 100 Hz). The monitor was positioned 40 cm in front of the pigeon’s right eye (Figure 1). The CRF center at each recording site was determined in the same way introduced in the previous study [46]. After that, the following types of stimuli were used to test the surround modulation properties of tectal neurons.

(1) Flashed stimuli. Flashed squares with different side lengths were used to map the size tuning curve of units at each recording site. Additionally, the diameters of CRF and eCRF were determined under this stimuli condition. The response magnitude continuously increased toward a peak value with each increase in stimulus size and then decreased and finally became asymptotic. The diameters of CRF (denoted by R_c_) was defined by the stimulus size at which the response reached a maximum [49], and the diameter of eCRF (denoted by R_e_) was determined by the largest size that reduced the peak neuronal response. Then, one side length of the flashed bars was kept fixed (set to R_c_ or R_e_), and the length of the other side varied in accordance with the previously mentioned flashed squares. Different sizes of squares or bars flashed at the center of the neuron’s CRF on a gray background in a pseudo-randomized order for 20 repetitions. Each stimulus lasted for 100 ms with an interstimulus interval of 100 ms. 

(2) Moving stimuli. The size of moving squares was the same as the above flashed ones. The motion direction was generally set as the one that evoked the highest mean firing rates. For most recording sites, we additionally tested with the motion direction orthogonal to the initial one to make a comparison and to further examine whether the result was dependent on the motion direction. The moving speed of each bar remained consistent (one pixel per frame, refresh rate of 100 Hz) for all motion paradigms. Note that the length is four times as long as R_c_ to assure that the neurons do not respond at the beginning and the end of each motion and the response remains stable throughout the experiment.

The signals were collected with a Cerebus system (Blackrock^®^ Microsystems, Salt Lake City, UT, USA) and amplified 4000×. The spikes were detected by thresholding the band-pass filtered (Second-order Butterworth) raw signals between 250 and 5 kHz with a sampling rate of 30 kHz. The threshold value was set to 5.25 times the standard deviation of the band passed signal (Figure 1). 

### 2.3. Data Analysis

**Figure 2:**
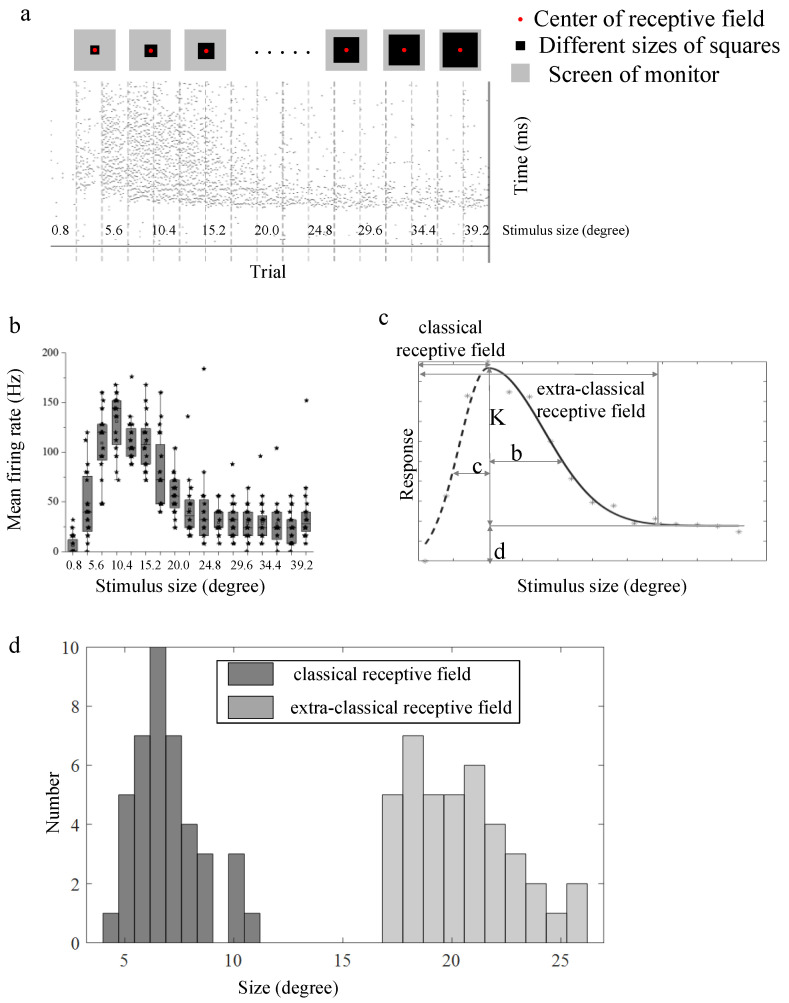
Size tuning curve of flashed squares stimuli as well as the estimated parameters. (**a**) Flashed squares (denoted by black square) of different sizes and the corresponding spike trains. The larger gray square indicates the scope of the screen. The middle red dot denotes the receptive field center; (**b**) the original mean firing rates of 20 repeats at an example recording site under each stimulus shown in (**a**). The horizontal line indicates the median of each group of data and the whiskers indicate the lowest and highest point within 1.5× the interquartile ranges of the lower or upper quartile, respectively. Each black asterisk represents firing rates of a single trial. (**c**) The fitted size tuning curve (adjust-R^2^ = 0.9462, left; adjust-R^2^ = 0.8628, right) of data shown in (**b**) and explanation of each fitting parameter as well as the indication of estimated classical receptive field and extra-classical receptive field size. *K* is the amplitude of the Gaussian function. *b* and *c* are widths. *d* is offset of asymptotic response from spontaneous activity. (**d**) statistical histogram of receptive classical receptive field size and extra-classical receptive field of all recorded neurons (*n* = 40).

Data analysis was performed in MATLAB R2019a (The MathWorks). The mean firing rate under each stimulus was first calculated. The size tuning curve (Figure 2c) based on mean firing rate was then fitted with the Piecewise Gaussian model [49] as follows,

(1) $$R(x)=\{\begin{array}{c} Ke^{-{\left(x-a\right)}^{2}/b^{2}}+d,x\geq a\\ (K+d)e^{-{\left(x-a\right)}^{2}/c^{2}},x\leq a \end{array}$$

where *x* is the stimulus size (side length of squares, length of the varying side of bars). *a* is the diameter of CRF. *K* is the amplitude of the Gaussian function. *b* and *c* are widths. *d* is offset of asymptotic response from spontaneous activity. *e* (Euler’s Number) is an irrational number and is the base of the natural logarithm. Generally, the size tuning curve was always fitted with the difference-of-Gaussians (DoG) model [7,50,51]. However, the data in our study were not consistent with this assumption. Thus, the data were divided into two phases, ascending and descending limbs. The parameters *K*, *b*, and *d* of the descending limb (solid line in Figure 2c) were first evaluated due to the greater number of sample points. The ascending limb of the size tuning curve (dotted line in Figure 2c) was then fitted using the fitted *K*, *d*, and the free parameter *c*. All values were optimized to minimize summation of the squared error to access a good fit, which was assessed using mean Adjust-R^2^. Each fitted size tuning curve was normalized to the maximum value.

The suppression index (SI) was finally calculated with *K*/(*K* + *d*) before the normalization to quantify the suppression strength. The index takes a value between 0 (indicates no suppression) and 1 (indicates complete suppression). The statistical analysis was carried out using the Wilcoxon signed-rank test for data of the same size and the Wilcoxon rank sum test for data of different sizes. The statistical comparison graphs were drawn using Origin 2019b.

## 3. Results

We recorded a total of 244 multi-units (Table 1) with an obvious CRF border (the estimated CRF from an example recording site were presented in Figure S1) from the middle layer OT of 17 pigeons. In total, 31 recording sites presenting unstable responses to repeat stimuli were discarded. Note that the recording sites from each pigeon were located in an approximate horizontal layer, thus their CRF centers were always separate. The surround modulation property could only be characterized for each recording site, respectively, and each would take about 3 h. As a result, only a limited number of recording sites (Table 1) could complete part of or all stimulation paradigms, including moving and flashed ones, due to the limited surviving time for the pigeon anesthetized with urethane. Finally, a total of 106 recording sites succeeded in carrying out all partial visual stimulation experiments (Table 2). Note that all those 106 recording sites have no motion direction selectivity. For each part of the following results, we only presented the detailed results of an example from a single recording site.

### 3.1. Surround Modulation Properties by Flashed Stimuli

Surround modulation properties were first tested with flashed squares of different sizes, located at their RF centers (indicated with red dots in Figure 2a). A total of 57 recording sites were examined in this paradigm (Table 2). About 70.2% (40/57) of recording sites presented surround suppression properties, that increased first and then inhibited as the flashed stimuli size grew larger (Figure 2b). The distribution of the estimated R_c_ and R_e_ (Figure 2d) derived from the fitting curve (Figure 2c) for those 40 recording sites showed that the size of eCRF was about 2–4 times as big as the CRF size. R_c_ is within the range of [4,5,6,7,8,9,10,11,12] degrees and R_e_ is within [17,18,19,20,21,22,23,24,25,26] degrees.

**Figure 3:**
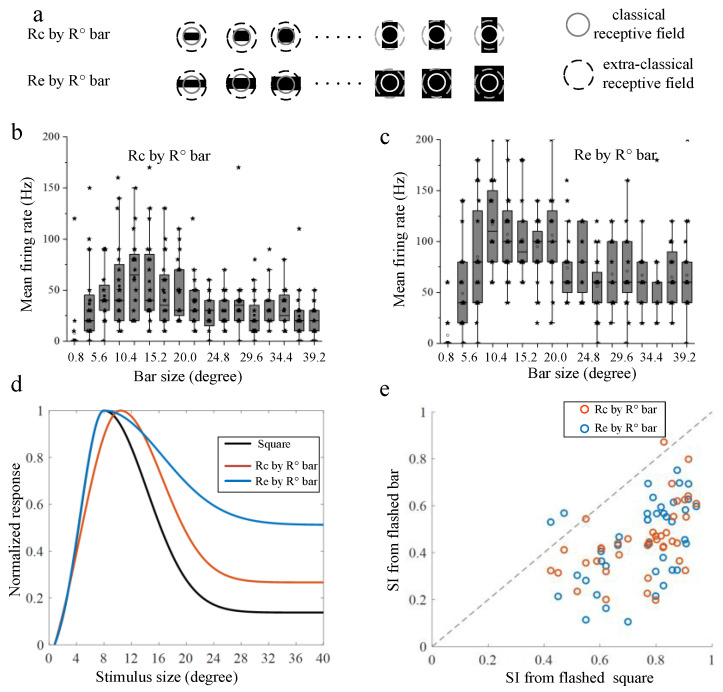
Summarized results of surround suppression induced by flashed bars and the statistical comparison with that by flashed squares. (**a**) Two groups of bars. One kept the fixed side length at R_c_ (R_c_ is the diameters of classical receptive field, upper), and another was set to R_e_ (R_e_ is the diameters of extra-classical receptive field, lower). The varying size lengths (indicated with R in (**b**–**e**)) were the same level of squares from 1 to 4 degrees. The solid line circle in each subfigure indicates the classical receptive field area and the dotted circle indicates the extra classical receptive field area, which is the same as in Figure 4a. The black square in each subfigure denotes varying lengths of flashed bars. (**b**,**c**) The mean firing rates of 20 repeats at an example recording site under each stimulus shown in (**a**); the horizontal line indicates the median of each group of data and the whiskers indicate the lowest and highest point within 1.5× of the interquartile ranges of the lower or upper quartile, respectively. Each black asterisk represents firing rates of a single trial. (**d**) The normalized fitted size-tuning curves under each stimulus shown in (**a**) (red: adjust-R^2^ = 0.9049, ascending limb; adjust-R^2^ = 0.8737, descending limb; blue: adjust-R^2^ = 0.9796, ascending limb; adjust-R^2^ = 0.8354, descending limb) as well as in Figure 2a (black: adjust-R^2^ = 0.9462, ascending limb; adjust-R^2^ = 0.8628, descending limb); (**e**) the suppression index for flashed square stimulus versus two groups of the flashed bars. Each dot represents SI from a single recording site. Red symbols show SIs of R_c_ by R bar, and blue of R_e_ by R bar. The diagonal line displays the locus of equal value.

**Figure 4:**
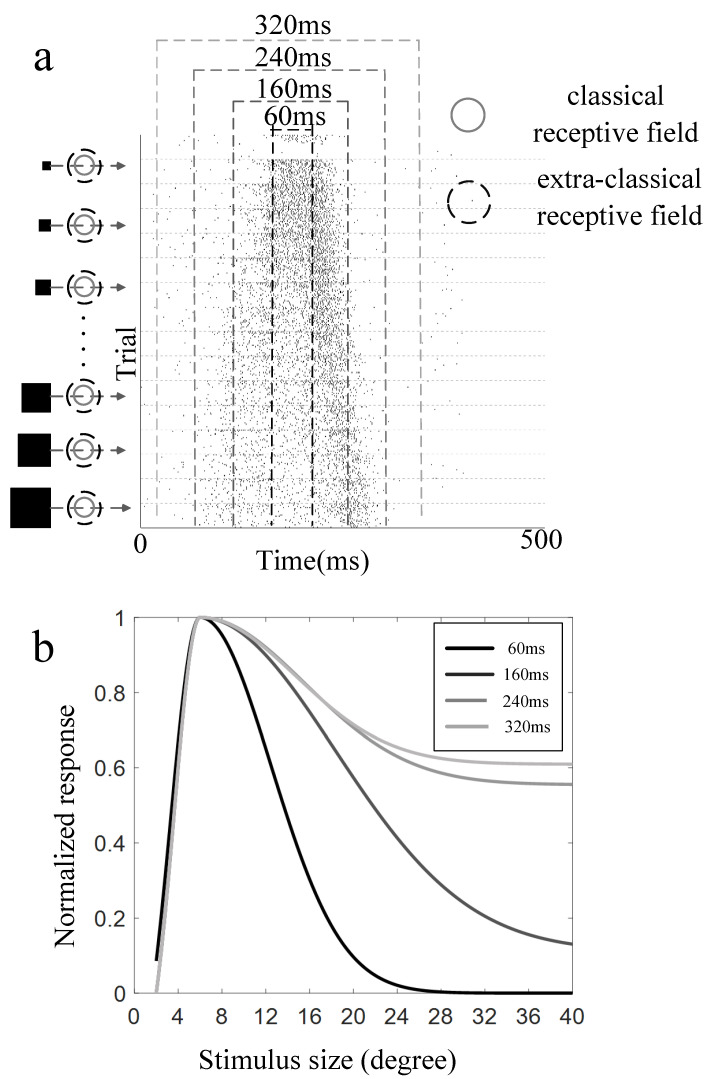
Summarized results of surround suppression by moving squares. (**a**) Visual stimuli of different sizes of moving squares, and their corresponding firing spike train of 20 repeats. The solid and dotted circle in each subfigure indicates the border of classical receptive field and extra classical receptive field, respectively. The black square beside each subfigure denotes the different sizes of moving squares. The pairs of dotted lines in different colors denoted different widths of the time window used to calculate the mean firing rate; (**b**) the size-tuning curves under different widths of the time window (60 ms: adjust-R^2^ = 0.9837, ascending limb; adjust-R^2^ = 0.9553, descending limb; 160 ms: adjust-R^2^ = 0.9796, ascending limb; adjust-R^2^ = 0.9819, descending limb; 240 ms: adjust-R^2^ = 0.8733, ascending limb; adjust-R^2^ = 0.9123, descending limb; 320 ms: adjust-R^2^ = 0.8925, ascending limb; adjust-R^2^ = 0.8815, descending limb).

Then, the surround modulation properties were tested with flashed bars, including two groups, one of which kept one side fixed at R_c_ (upper in Figure 3a), whereas in the other group it was fixed at R_e_ (lower in Figure 3a). The varied side lengths in each group of bars were set at the same level as squares. In Figure 3a, the solid line circle indicates the border of the CRF and the dotted circle indicates that of the eCRF (the same as in Figure 4a). The black square denotes different sizes of bars. Similar to the surround modulation pattern obtained by the flashed squares, the response increases first and then decreases as the varying side length of each group of bars grew larger (Figure 3b,c). The size-tuning curves were fitted similarly. The normalized tuning curve compared with that under the flashed square condition (Figure 3b) showed that the size of stimuli that evoked the maximum response was similar among all three curves, and the suppression degrees by larger stimuli were very different from each other. The calculated SIs derived from the two groups of flashed bars were compared with those from the flashed squares. As shown in Figure 3c, SIs of flashed bars were, respectively, plotted against those of flashed squares for all selected recording sites (*n* = 40). Note that red dots in Figure 3c indicated results of flashed bars with one side fixed at R_c_ and blue dots indicated those fixed at R_e_. From Figure 3c, we can see that most dots (39 out of 40 for R_c_ and 38 out of 40 for R_e_, Wilcoxon signed-rank test, *p* < 0.001) were below the diagonal line, indicating much stronger surround suppression resulting from flashed squares than from flashed bars. There was no significant difference between surround suppression induced by the two groups of bars (Wilcoxon rank sum test, *p* > 0.05). The results suggested that suppression was more intense when tectal neurons received global full surrounding stimulation than local partially surrounding stimulation along one direction, no matter whether the short side of the bar only covered the CRF of tectal neurons or extended to their eCRF. 

### 3.2. Surround Modulation Properties by Moving Stimuli

Surround modulation properties were further tested with moving squares and bars. A total of 64 recording sites were tested in the moving paradigm. About 71.9% (46/64) presented suppression properties by surrounding moving stimuli. In total, 15 out of the 46 recording sites were tested in both flashed and moving paradigms. 

Since the highest firing rate was elicited when the stimulus crossed the RF border, there was a skewed response offset as the stimulus grew larger (Figure 4a). The mean firing rate within different time windows (60, 160, 240, and 320 ms, dotted rectangles in Figure 4a, centered at the CRF center) along the moving path were calculated and further used to map the size tuning curves, respectively. The comparison of the normalized size tuning curves derived from different time windows (Figure 4b) showed that the suppression grew weaker as the time window grew larger, suggesting the surround suppression induced by moving stimuli was the strongest when the stimuli just passed near the CRF center. Note that the width of each time window was manually adjusted based on their firing patterns to find a distinct modulation pattern and the moderate the difference between modulation degrees based on different time windows. The other sets of time windows were also tried, and some of them showed similar results (see Figure S2 in Supplementary Materials), but not all of them. We think it may depend on the structure of excited CRF and the inhibited surrounding eCRF, the latter of which is not necessarily an exact multiple of the former for each recording site. The RF structural properties of tectal neurons were further discussed in the Section 4.

In addition, other motion directions were also used to guarantee the independence of our results on the direction of motion. The comparison results showed that there was no significant difference between surround modulation properties derived from different motion directions, albeit the response patterns to different motion directions were a little different. Detailed results for the other directions were given in the (Supplementary Material Figure S3).

### 3.3. Comparison of Surround Suppression by Moving and Flashed Stimuli

**Figure 5:**
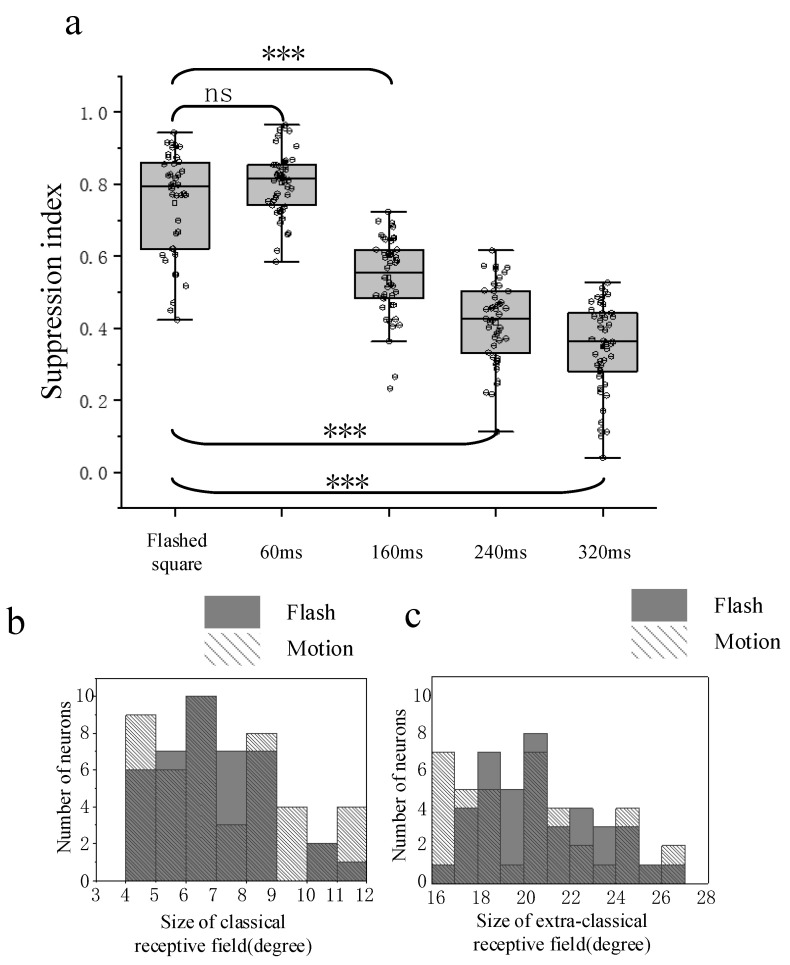
Statistical comparison of surround suppression by flashed and moving stimuli. (**a**) The boxplot graphs of suppression indexes from the moving square stimulus of different widths of the time window and to the flashed square stimulus. The horizontal line indicates the median of each group of data and the whiskers indicate the lowest and highest points within 1.5× the interquartile ranges of the lower or upper quartile, respectively. The ‘ns’ indicates no significant difference between two groups of data (Wilcoxon rank sum test, *p* > 0.05) and ‘***’ indicates a significant difference between two groups of data (Wilcoxon rank sum test, *p* < 0.001). (**b**) Distribution of classical receptive field size for all recorded sites estimated in flashed and moving conditions, respectively. Gray filled rectangle indicates results in flashed conditions, while texture filled rectangle indicates those in moving conditions. (**c**) Distribution of extra classical receptive field size for all recorded sites estimated in flashed and moving conditions, respectively. Gray filled rectangle indicates results in flashed conditions, while texture filled rectangle indicates those in moving conditions.

The surround suppression properties obtained from flashed (40 recording sites) and moving stimuli (46 recording sites) conditions were finally compared. The calculated SI based on response within different time windows in the moving paradigm was receptively compared with those under flashed stimuli (Figure 5a). The comparison results showed that the surround suppression derived from moving stimuli was not significantly different from that from flashed stimuli when the time window used to quantify response was short, corresponding to when the squares just moved near the CRF center. However, the suppression degree became weaker than that in the flashed condition and moving condition with a smaller response time window (Wilcoxon rank sum test, *p* < 0.001) when the time window was set a little larger, in which case the response to stimuli which moved across the CRF border was also taken into account. Note that when a square moves across the CRF boundary, the neuron just received local partially surrounding stimulation. In such a condition, the response ought to be a litter larger, resulting in weaker surround suppression, which was consistent with the phenomenon found in the flashed stimuli condition.

Furthermore, the CRF and eCRF sizes obtained from flashed conditions were compared with those from moving stimuli (Figure 5b,c), showing that there was no significant difference between the results of the two stimuli conditions (Wilcoxon rank sum test, *p* > 0.05).

## 4. Discussion

**Figure 6:**
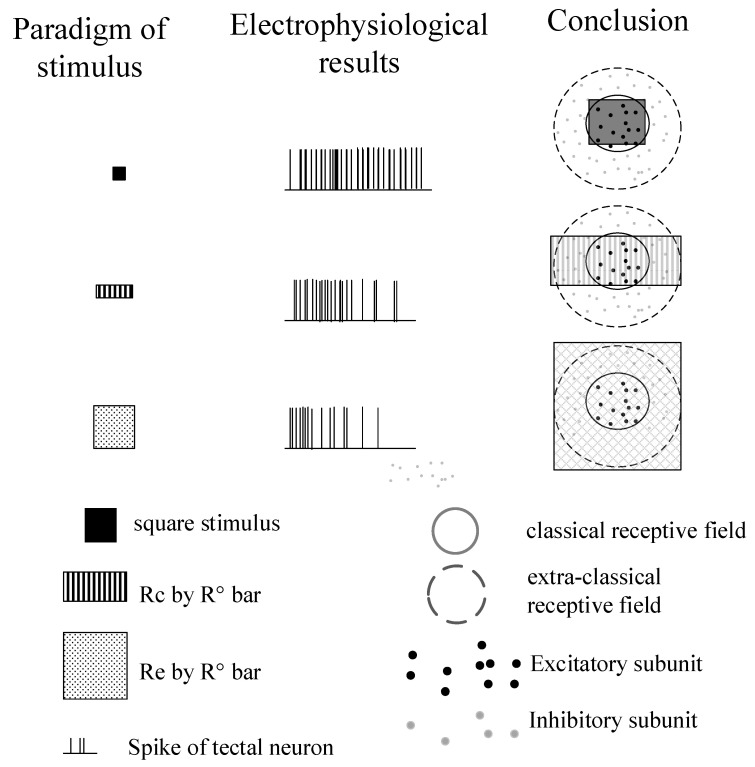
Illustration of data and conclusions. Tetcal response (middle subfigure to different sizes of flashed squares and bars (left subfigure) suggest that inhibitions performed on tectal neurons appear to be full surrounding rather than locally lateral (right subfigure). The solid line circle in each subfigure indicates the classical receptive field area and the dotted circle indicates the extra-classical receptive field area.

The results showed that most tectal responses presented suppression as the size of the stimuli grew larger in both flashed and moving conditions. In addition, the suppression was more intense when the surrounding of CRF was fully stimulated compared to partially stimulated along any motion direction, suggesting inhibitions performed on tectal neurons appear to be full surrounding rather than local lateral. The results (Figure 6) provide possible hypotheses about the arrangement of inhibitions from other nuclei.

Our results showed that the larger moving and flashed stimuli modulated most tectal neurons in pigeons in a similar way. Responses to both moving and flashed squares presented as suppressive rather than facilitative, which were first enhanced and then suppressed as stimuli grew larger. The modulation trend was consistent with previous reports on mammals and birds. What is more, we found the following findings to be novel. The suppression degree induced by flashed squares was not significantly different from that by moving squares when it crossed near the cell’s CRF center. The suppression degree grew weaker when considering the response to moving stimuli crossing the CRF border together. What is more, the surround suppression induced by bars was weaker than that by squares.

Studies on surrounding modulation properties were always accompanied by the measurement and statistics of CRF and eCRF, both of which are collectively referred to as RF. The RF in pigeon OT was firstly marked in the 1970s [38,52], and the existence of the center-surround structure was tested by using wider moving or flashing bars than the excitatory area of the field in the subsequent studies [40,53,54]. Prior studies have noted that the RFs of tectal neurons increased in size and their shapes became more complex from the superficial to the deep layers [53]. The CRF in the intermediate layers exhibited a general size range of 5–10 degrees, while eCRF had a range of 10–27 degrees [55]. The RFs of tectal units were mostly round or oval-shaped [53] and characterized by a concentric organization consisting of an excitatory RF surrounded by an inhibitory RF. These properties are also in accordance with our findings. However, these earlier studies just reported a standard (center-surround) structure of RFs of tectal neurons by simply using moving or flashing bars, but there lacks a quantitative comparison between them. Recent evidence suggested that visual neurons in the dorsal, dorso-lateral, lateral, and ventro-lateral tectum were different from those in the ventral tectum in their RF organization, visual responses, and laminar locations [55]. The RF of neurons in our recording location (dorso-lateral tectum) was also composed of an excitatory center and an inhibitory surround, in agreement with the standard structure of RFs reported previously [38,40,41,53]. What is more, we further found that the surround suppression induced by squares extending outside the CRF was significantly larger than that by bars with only one side extending outside the CRF. These results suggest inhibitions performed on tectal neurons appear to be full surrounding rather than local lateral (Figure 6). The results implied a potential spatial arrangement of inhibitions existing in OT coming from other layers or nuclei. However, the conclusion still requires further confirmation. Nevertheless, early studies have speculated that the inhibition may be from the retina and transmitted to the tectal cells by way of both feedforward and feedback pathways [56,57,58]. Excitatory and inhibitory CRF of tectal cells were differentially modified by magnocellular and parvocellular divisions of the pigeon nucleus isthmi [48,59]. In birds, the tectum and nucleus isthmi form the midbrain network [60,61], which involves visual saliency representation and allows efficient information encoding. Meanwhile, visual saliency is the neural basis of figure–ground segregation, visual–target detection. Taken together, the findings of this paper would help to understand the mechanism of target detection in dynamic scenes. 

Note that several earlier studies have reported that most tectal neurons were sensitive to moving stimuli but did not respond to stationary ones, and only a small number of tectal neurons responded to both moving and flashed stimuli [38,42,52,62]. However, in our study, we found lots of the latter kind of neurons, and made a further comparison upon surround suppression induced by moving stimuli and by flashed ones. The unicity of the recorded neuronal response may be due to our relative fixed recording site, the dorso-lateral tectal section that was easily accessed by a simple craniotomy [47]. The recording site and the corresponding neuronal response were quite consistent with those earlier reports [55].

Additionally, the surround modulation properties were explored in another way by other researchers using groups of bars with different types of center-surround contrast, including moving direction, bar orientation, and luminance [44,45,46]. The results showed that tectal neuronal response was suppressed when the visual stimuli of the surroundings were consistent with that of the center. Meanwhile, the tectal neuronal response was popped out when the contrasts between center and surround were moving direction and luminance [44,46]. Most recent work has also compared the suppression induced by spatial contrast based on luminance and moving direction, which represented the static and motion information of a visual object, respectively, and showed that suppression resulting from motion direction contrast was smaller than that from luminance contrast [46]. The results were quite consistent with ours, which showed that the suppression induced by flashed stimuli was stronger than that by moving stimuli when the time window used to measure neuronal response was large.

Taken together, visual neurons in both avian OT and the preliminary stages of the visual system of mammals, including retina [63,64,65,66], SC [22,67], LGN [33,68] and V1 [32,69,70], have similar RF structures, most of which presented a concentric organization characterized by an excitatory CRF surrounded by an inhibitory eCRF. The main difference between birds and mammals is that most V1 neurons are orientation selective which means that they respond best to particular orientation stimuli but not to the orthogonal orientation [71]. The spatial distribution of surround suppression in V1 was nonuniformly distributed [20]. The results of our study also showed that surround suppression was more intense when the surrounding of CRF of tectal neurons was fully stimulated compared to partially stimulated, which may be used to explain the habitation of birds who are more likely to focus on small targets in a cluttered scene. Our study supplemented the existing studies on surround modulation properties of visual neurons in both avians and mammals and deepened our understanding of information transmission rules in the avian midbrain. Nevertheless, there were still some limitations to our study, particularly that our conclusion was drawn from urethane-anesthetized pigeons. Albeit urethane has minimal effects on synaptic transmission compared with other anesthetics [72,73], the interaction among neurons might be affected. Thus, it would be interesting to further examine the results in awake avians.

## 5. Conclusions

We thoroughly studied surround modulation properties of tectal neurons in pigeons. The results showed that most neurons presented similar surround suppression properties both in flashed and motion paradigms. Both responses were first enhanced and then suppressed as the size of stimuli increased. The property was consistent with those found in mammals V1, LGN, and SC with drifting sinusoid gratings as stimuli. Another finding that full surround suppression was stronger than partially surround suppression was novel and complemented the existing studies. Furthermore, this study provides a possible hypothesis upon the spatial arrangement of lateral inhibitions from feedback or feedforward streams, which would deepen our understanding of visual information transmission in the tectofugal pathway of avians. In addition, the stronger full surround suppression, corresponding to global inhibition [74], can enhance the saliency degree of visual objects and may help animals to detect small objects more easily, which is very important for their survival in a complex natural environment.

## Figures and Tables

**Table 1:** The number of effective recording sites and sites for the analysis of each pigeon.

**Pigeon ID**	**#1**	**#2**	**#3**	**#4**	**#5**	**#6**	**#7**	**#8**	**#9**	**#10**	**#11**
Effective recording sites	14	15	15	16	12	14	14	12	15	13	16
Carrying out further visual stimulation experiments	6	9	5	10	4	5	6	4	8	3	5
**Pigeon ID**	**#12**	**#13**	**#14**	**#15**	**#16**	**#17**	**Total**				
Effective recording sites	14	15	16	16	15	12	244				
Carrying out further visual stimulation experiments	6	8	6	7	5	9	106				

**Table 2:** The number of those who completed the whole visual stimulation experiments, or partial.

Stimulation Conditions	Carrying Out Further Visual Stimulation Experiments	Selected for Statistical Comparison under Each Stimulation Condition	Used for Statistical Comparison between Moving and Flashed Stimuli
Flashed stimuli	57	40	40
Motion stimuli	64	46	46
Both types	15	15	15

## Data Availability

The datasets analyzed in the current study are available from the corresponding author on reasonable request.

## Supplementary Materials

The following supporting information can be downloaded at: https://www.mdpi.com/article/10.3390/ani12040475/s1, Figure S1: The receptive field area of example recording sites; Figure S2: The size-tuning curves under different widths of the time window; Figure S3: An example result of surround suppression by moving squares in a different moving direction.
