# Supplementary material for: Surround Modulation Properties of Tectal Neurons in Pigeons Characterized by Moving and Flashed Stimuli
Source: Animals (Basel). 2022 Feb 15;12(4):475. doi: 10.3390/ani12040475 (PMC8868286; doi:10.3390/ani12040475)

Figure S1. The receptive field area of example recording sites. (a) The spatial receptive field in each time intervals after the onset of stimuli. (b) The spike train corresponding to each single location. The left one subfigures show the receptive field area (left) estimated with sparse noise flashed. The right five subfigures show ten repeats of the firing spike train corresponding to the location indicated with the same color. The first line indicates the onset of each stimulus and the second denotes the offset.

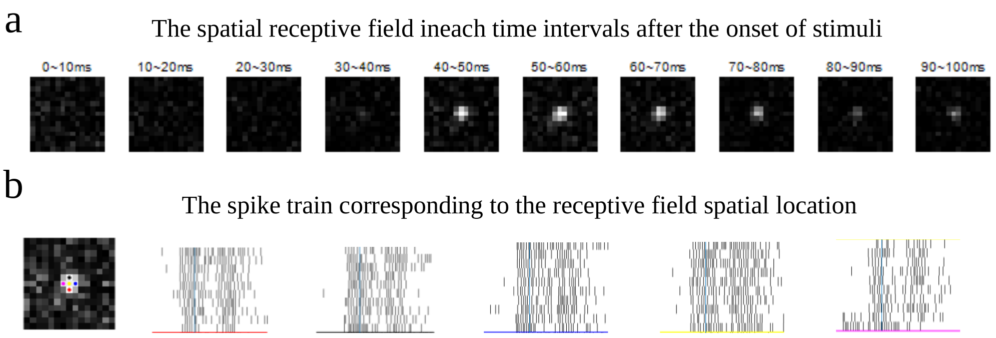

Figure S2. The size-tuning curves under different widths of the time window. (40ms: adjust-R2=0.9757, ascending limb; adjust-R2=0.9428, descending limb; 100ms: adjust-R2=0.9574, ascending limb; adjust-R2=0.9514, descending limb; 120ms: adjust-R2=0.8911, ascending limb; adjust-R2=0.9236, decending limb; 180ms: adjust-R2=0.9215, ascending limb; adjust-R2=0.8688; 280ms: adjust-R2=0.8714, ascending limb; adjust-R2=0.8632, descending limb).

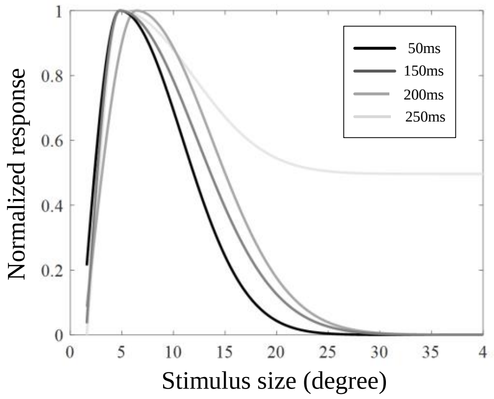

Figure S3. An example result of surround suppression by moving squares in another different moving direction. (a) Visual stimuli of different size moving square, as well as their corresponding firing spike train in 20 repeats. The solid line circle in each subfigure indicates the classical receptive field area and the dotted circle indicates the extra classical receptive field area. The black square beside each subfigure denotes varying sizes of the moving square. The pairs of dotted lines in different colors denoted different widths of the time window in which the mean firing rate was calculated. (b) The size-tuning curves under different widths of time window. (60ms: adjust-R2=0.9867, ascending limb ; adjust-R2=0.9325, descending limb ; 160ms: adjust-R2=0.9778, ascending limb ; adjust-R2=0.9592, descending limb ;

240ms: adjust-R2=0.9817, ascending limb; adjust-R2=0.9447, descending limb ; 320ms: adjust-R2=0.9827, ascending limb ; adjust-R2=0.9246, descending limb).

a

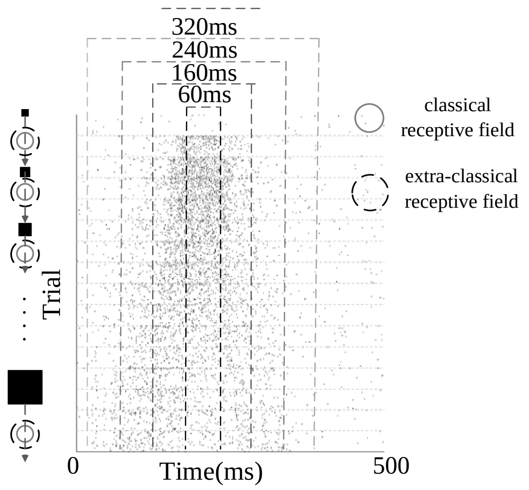

b

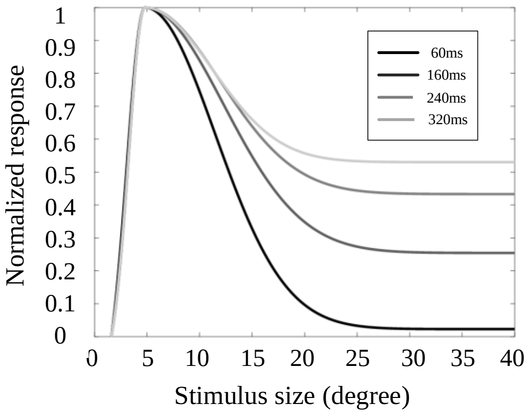

Supplement: Supplementary file 1 [file animals-12-00475-s001.zip › animals-1512962-supplementary.pdf]
